# A Prospective Study of the Incidence of Retinopathy of Prematurity in China: Evaluation of Different Screening Criteria

**DOI:** 10.1155/2016/5918736

**Published:** 2016-06-13

**Authors:** Qiuping Li, Zonghua Wang, Ruijuan Wang, Hongyi Tang, Haihua Chen, Zhichun Feng

**Affiliations:** ^1^Neonatal Intensive Care Unit, BaYi Children's Hospital of The General Military Hospital of Beijing PLA, Beijing 100700, China; ^2^Department of Ophthalmology, The Military General Hospital of Beijing PLA, Beijing 100700, China

## Abstract

To investigate the incidence of Retinopathy of Prematurity (ROP) in Beijing, North China, and to evaluate the effectiveness of different ROP screening criteria, we conducted a prospective cohort study in a single-neonatal intensive care unit (NICU). A total of 2997 premature infants with birth weight (BW) ≤ 2000 g and/or gestational age (GA) ≤ 34 weeks had completed ROP screening. ROP was diagnosed in 356 (11.9%) infants. The mean GA was 30.46 ± 1.98 weeks and the mean BW was 1477.35 ± 371.29 g. Of the 59 (2.0%) infants receiving treatment, the mean GA was 29.37 ± 2.10 weeks, and the mean BW was 1240.80 ± 330.71 g. The incidence of ROP declined from 14.7% in 2009 and 11.1% in 2010 to 9.5% in 2011. The United Kingdom (UK) criteria could reduce the screening number by 40.8%, and 3 infants with type I ROP needing treatment were missed, but none in 2011. The United States (US) criteria could reduce the screening number by 66.5%, and 10 infants with type I ROP needing treatment were missed, including one in 2011. So the UK criteria may be appropriate for screening of ROP in our NICU in 2011. Future multisite epidemiologic studies are required to establish suitable ROP screening criteria in China.

## 1. Introduction

Retinopathy of Prematurity (ROP) is one of the most common eye disorders in premature infants, characterized by abnormal proliferation of retinal blood vessels. If untreated, severe ROP can lead to retinal detachment and blindness. With the development of perinatal health care, the incidence of ROP has greatly declined in the developed countries. ROP is mainly found in premature infants with a gestational age of <28 weeks and birth weight <1500 g, and the incidence of ROP requiring treatment is low in the developed countries [1, 2]. However, for developing countries such as China, India, Turkey, Brazil, and Vietnam, the incidence of ROP remains high [3, 4], especially since the number and survival rate of premature infants with low birth weight have increased in the neonatal intensive care unit (NICU) after introduction of new techniques such as mechanical ventilation and use of surfactants [5]. It has been reported that ROP leads to blindness in approximately 50,000 children per year worldwide [5]. In China, a developing country that has the largest population in the world, approximately 16 million infants, of which 1.5 million are premature infants, are born every year. ROP remains a significant public health problem for premature infants in China. Indeed, several studies have reported that ROP is a leading cause of blindness in Chinese children [6, 7]. However, to date, a large epidemiologic study of the incidence of ROP in China has not been performed.

Appropriate screening and timely treatment are important to avoid ROP-induced blindness. Because the ROP screening procedure can cause pain in neonates and has medical costs, appropriate screening criteria should be applied to minimize the number of neonates for screening without missing any type I ROP that obviates treatment [8]. It is well known that the low gestational age, low birth weight, and prolonged use of oxygen are major risk factors for the development of ROP, and therefore most screening criteria for ROP are made based on the gestational age (GA), birth weight (BW), and use of oxygen. For example, after several revisions, the current ROP screening criteria in the United States (US) included GA ≤ 30 weeks and/or BW ≤ 1500 g [9]. The ROP screening criteria in the United Kingdom (UK) included GA ≤ 32 weeks and/or BW ≤ 1500 g [10]. In China, the ROP screening guideline, which was recommended by the Ministry of Health in 2004, includes GA ≤ 34 weeks and/or BW ≤ 2000 g [11]. However, the criteria may not be suitable for the current ROP screening status, since neonatal care has been greatly improved during the past 10 years and may affect incidence of disease, which can affect the positive predictive value (PPV). A prospective study undertaken in two tertiary hospitals in Shanghai, Southern China, demonstrates that the screening criteria used in the US and UK may not suitable for China, and narrower criteria including GA ≤ 33 weeks and or BW ≤ 1750 g reduce screening number by 16.9% without any infants missing treatment [12]. Since neonatal care varies greatly in different regions of China and ROP incidence differs among different centers, it is necessary to examine whether these criteria are also applicable to other regions of China.

In this prospective study, we analyzed the incidence of ROP between 2009 and 2011 in the largest tertiary level NICU in Beijing, North China. The purpose of this study was to evaluate the effectiveness of different international ROP screening guidelines.

## 2. Materials and Methods

### 2.1. Patients

The Medical Ethics Committee of BaYi Children's Hospital of the Military General Hospital of Beijing approved this study. All patients' parents or legal guardians gave their informed consent prior to their inclusion in the study. This prospective study initially included 3095 consecutive premature infants, who underwent ROP screening at the NICU of our hospital between January 1, 2009, and December 31, 2011. The inclusion criteria were as follows: (1) infants with BW ≤ 2000 g and/or GA ≤ 34 weeks and (2) infants with BW > 2000 g and or GA > 34 weeks who underwent invasive mechanical ventilation for more than one week or continuous supplemental oxygen therapy for more than two weeks. The exclusion criteria were as follows: (1) death before the initial screen; (2) death or loss to follow-up before complete retinal vascularization was developed; (3) incomplete screening procedure; and (4) infants who underwent ROP screening at other hospitals.

All infants underwent the first examination at 4–6 weeks after birth or 32–34 weeks of corrected GA. Infants with lesions less than prethreshold ROP were followed up every two weeks. Infants with type II prethreshold ROP or retinal vascularization within zone I were followed up every week until the peripheral retinal vascularization was complete or the lesions were resolved.

### 2.2. Ophthalmologic Examinations

Prior to the examination, infants were deprived of food and water for 2 h. Infant pupils were dilated with mydriatic eyedrops (0.5% tropicamide, 3-4 times for 10 min per time), followed by application of local anesthetic eye drops (0.4% oxybuprocaine). Ofloxacin (0.3%) was applied for contact between the camera lens and the cornea. Ophthalmologic examination was performed at the NICU using the RetCam II digital camera (Clarity Medical systems, Inc., USA). Infants were carefully observed for 2 h. ROP was analyzed according to criteria set by the international classification of Retinopathy of Prematurity by skilled ophthalmologists [13]. For patients undergoing multiple ophthalmologic examinations, the most severe result was used for analysis. Screening number reduction (%) = infants not required for screening/2997 × 100%.

### 2.3. Treatment

Laser photocoagulation or intravitreal injection of ranibizumab was performed within 72 h after diagnosis of type I ROP. The diagnosis of type I ROP and type II ROP was made according to the criteria set by the Early Treatment for Retinopathy of Prematurity Cooperative Group [24], as follows: (1) type I ROP: (a) zone I, any stage with plus disease; (b) zone I, stage 3, with or without plus disease; (c) zone II, stage 2 or 3, with plus disease; (2) type II ROP: (a) zone 1, stage 1 or 2, without plus disease; (b) zone 2, stage 3, without plus disease.

### 2.4. Statistical Analysis

Statistical analyses were performed using SPSS software (version 18.0, Chicago, USA). Quantitative data are expressed as means ± standard deviation. Student's *t*-tests and one-way analysis of variance (ANOVA) were used to compare the difference between two or more than two groups, respectively. Categorical data were analyzed using chi-square tests. Probability values less than 0.05 were considered statistically significant.

## 3. Results

### 3.1. Baseline Characteristics

This study initially included 3095 consecutive premature infants undergoing ROP screening between January 1, 2009, and December 31, 2011. In 2009, 2010, and 2011, 1038, 1208, and 849 infants were screened for ROP, respectively. Ninety-eight infants were excluded from the study, including 37 (3.56%) in 2009, 33 (2.73%) in 2010, and 28 (3.30%) in 2011. Fifty infants (19 in 2009, 17 in 2010, and 14 in 2011) failed to complete the ROP screening procedure due to death or giving up treatment. In addition, 48 infants (18 in 2009, 16 in 2010, and 14 in 2011) were lost to follow-up because their parents transferred them to other hospitals before the completion of the screening process. Finally, 2997 infants (1746 males and 1251 females) were included in this study. The average GA was 31.90 ± 1.91 weeks (range, 24–36 weeks). The average BW was 1814.54 ± 445.17 g (range, 680–3700 g). 1415 infants were delivered vaginally, and 1578 infants were delivered by cesarean section. 2343 cases had singleton gestation births, and 654 cases had multiple gestation births. Of the 2997 infants with complete ROP screening, 356 (11.9%) infants had ROP, and 59 (2.0%) infants received treatments, including 54 (1.8%) with laser photocoagulation and 5 (0.2%) with intravitreal injection of ranibizumab.  Table 1 summarizes the baseline characteristics of ROP and non-ROP infants. There were no significant differences in gender and singleton or multiple gestation births between the two groups (*p* > 0.05). ROP infants had a significantly lower GA and BW compared with non-ROP infants (*p* < 0.001). The percentage of those who required supplemental oxygen therapy, asphyxia at birth, septicemia, bronchopulmonary dysplasia (BPD), blood transfusion, neonatal respiratory distress syndrome (NRDS), apnea, and assisted ventilation was significantly higher in the ROP infants compared with non-ROP infants (*p* ≤ 0.001).

### 3.2. Yearly ROP Incidence Rate

**Figure 1:**
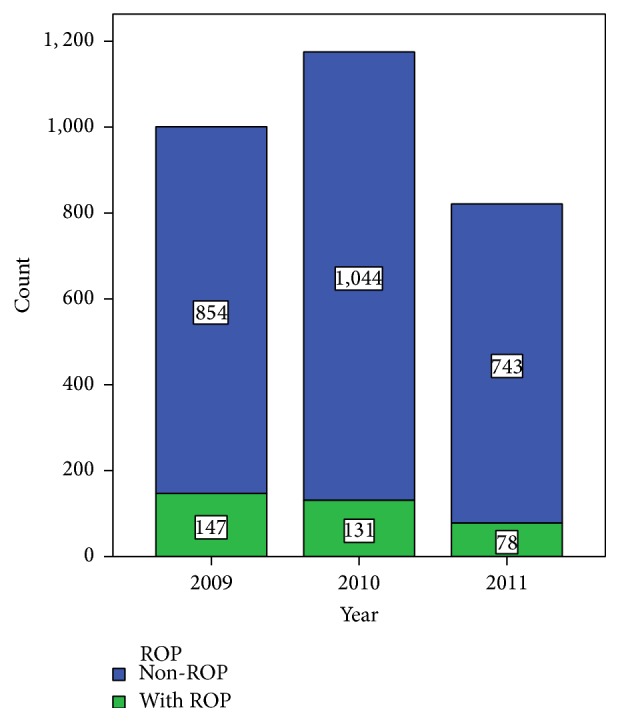
The number of ROP infants and ROP incidence in 2009, 2010, and 2011.

**Figure 2:**
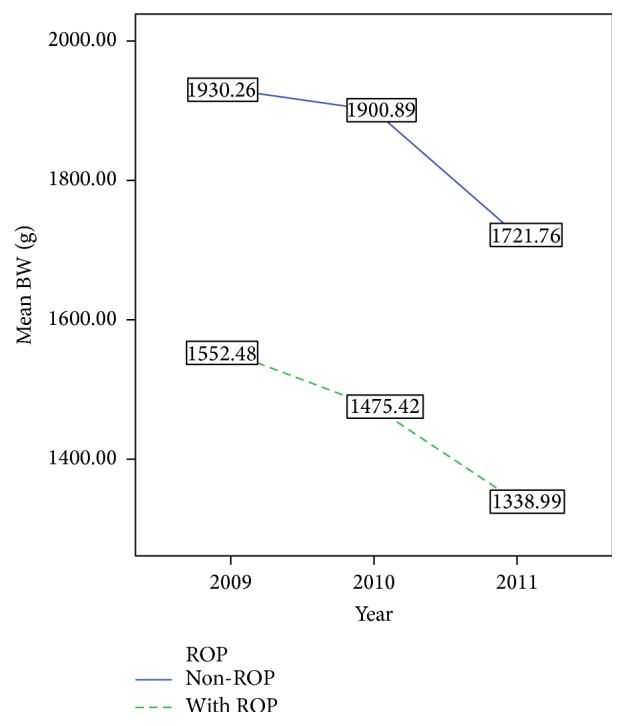
The mean birth weight of ROP and non-ROP infants in 2009, 2010, and 2011.

**Figure 3:**
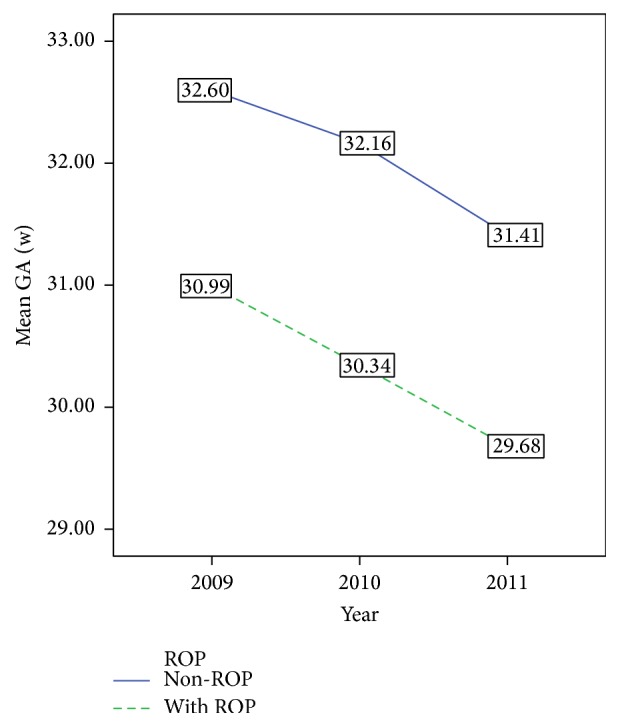
The mean gestational age of ROP and non-ROP infants in 2009, 2010, and 2011.

**Figure 4:**
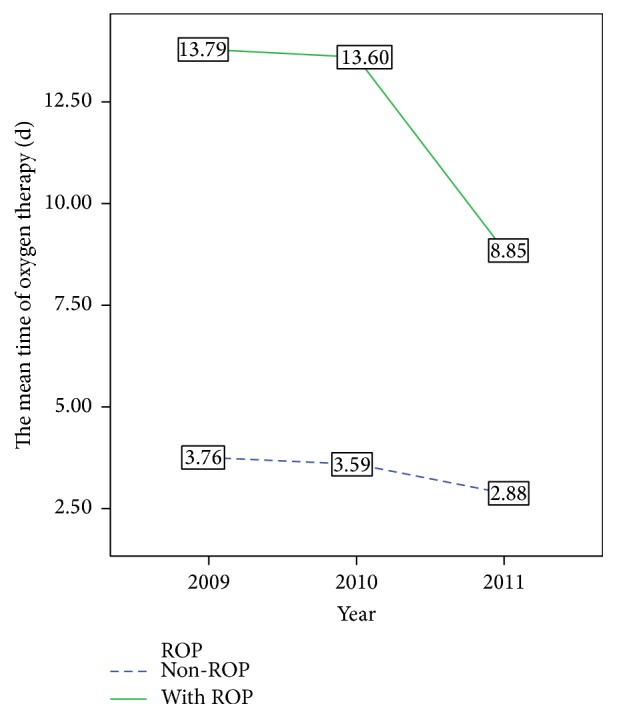
The mean time for continuous oxygen therapy of ROP and non-ROP infants in 2009, 2010, and 2011.

In 2009, 2010, and 2011, 1001, 1175, and 821 infants were screened for ROP, respectively. ROP was found in 147 (14.7%) infants in 2009, in 131 (11.1%) infants in 2010, and in 78 (9.5%) infants in 2011 (Figure 1). The yearly incidence rate was significantly decreased from 2009 to 2011 (*χ* ^2^ = 12.566, *p* < 0.01). The mean BW and GA of both ROP and non-ROP infants also decreased from 2009 to 2011 (Figures 2 and 3). In addition, the mean time for continuous oxygen therapy for all infants was 5.2 days (median 3, IQR 0–7) in 2009, 4.7 days (median 3, IQR 0–7) in 2010, and 3.4 days (median 0, IQR 0–4) in 2011. The mean time for continuous oxygen therapy for ROP infants was 13.7 days (median 9, IQR 4–17) in 2009, 13.6 days (median 11, IQR 5–17) in 2010, and 8.85 days (median 2, IQR 0–15) in 2011. Furthermore, the time for continuous oxygen therapy for non-ROP infants was 3.76 days (median 2, IQR 0–5) in 2009, 3.59 days (median 2, IQR 0–6) in 2010, and 2.88 days (median 0, IQR 0–3) in 2011. For both ROP and non-ROP infants, the time for continuous oxygen therapy was significantly decreased from 2009 to 2011 (*p* < 0.05, Figure 4). These findings suggest that the yearly ROP incidence rate decreased over time despite a decrease in the BW, GA, and the time for continuous oxygen therapy.

### 3.3. ROP Incidence Rate according to GA and BW

**Figure 5:**
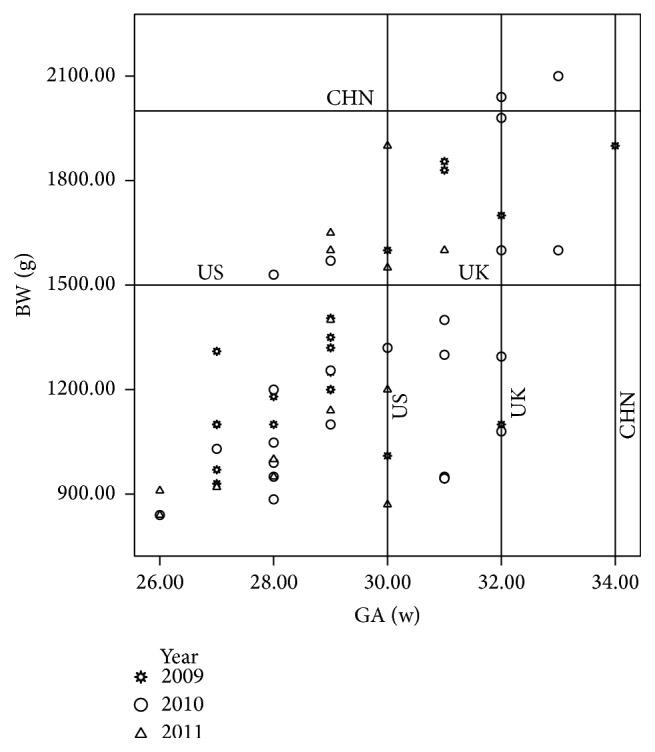
The distribution of type I ROP needing treatment according to the GA and BW in 2009, 2010, and 2011.

Tables 2 and 3 summarize the incidence rates of ROP with different stages and zones according to the GA and BW. The incidence rate was increased with the decrease in the GA and BW. Of the 168 infants with a GA ≤ 28 weeks, 60 (35.7%) infants had ROP, and 21 (12.5%) infants had type I ROP resulting in treatment. Of the 70 infants with BW ≤ 1 kg, 32 (45.7%) infants had ROP, and 15 (21.4%) infants had type I ROP. Of the 820 infants with BW ≤ 1.5 kg, 207 (25.2%) infants had ROP, and 42 (5.2%) infants had type I ROP. Of the total 356 ROP infants, 59 (16.6%) infants had type I ROP and received treatment, and 19 (5.3%) infants had acute progressive ROP (AP-ROP). There were 21, 24, and 14 infants with type I ROP who required treatment in 2009, 2010, and 2011, respectively. The distribution of type I ROP according to GA and BW is shown in Figure 5. In addition, there were 15, 10, and 3 infants with type II ROP in 2009, 2010, and 2011, respectively.

### 3.4. Evaluation of Different Screening Criteria for ROP

Table 4 summarizes the effect of different screening criteria for ROP on screening number and missed cases. If the screening criteria included both GA ≤ 34 weeks and BW ≤ 2000 g, 987 infants did not require screening. This resulted in a reduction of the screening number by 32.9%. Only two infants with type I ROP which required treatment were missed in 2010. If the screening criteria included GA ≤ 33 weeks and/or BW ≤ 1750 g, 588 infants did not require screening. This resulted in a reduction of the screening number by 19.6%. Only one infant who required treatment was missed in 2009. According to the UK criteria (GA ≤ 32 weeks and/or BW ≤ 1500 g), 1222 infants did not require screening, thus reducing the screening number by 40.8%. Fifty-three ROP infants were missed including three infants with type I ROP. In 2011, only three infants with ROP were missed, but none of them had type I ROP. According to the US criteria (GA ≤ 30 weeks and/or BW ≤ 1500 g), 1922 infants did not require screening; thus the screening number was reduced by 66.5%. However, 118 ROP infants were missed including ten infants with type I ROP. In 2011, one infant with type I ROP was missed.

## 4. Discussion

ROP is a main cause of treatable blindness in children in China. In 2004, Liu et al. reported that ROP caused blindness in 67 (37.9%) of 177 blind students in a blind school in Guangzhou [25]. Similarly, in 2005, Ji and Shen reported that ROP as the leading cause of blindness caused blindness in 32 (32.98%) of 97 blind students in a blind school in Shanghai [7]. Therefore, appropriate ROP screen guidelines are important for screening of ROP infants to avoid blindness in children. In the present study, we analyzed different ROP screening criteria based on examination of 2997 infants in a tertiary level NICU in Beijing, China. We found that if the UK screening criteria were adopted, the screening number was reduced by 40.8%, and only three infants with type I ROP that required treatment were missed. None of the three infants with type I ROP were reported in 2011. Even though the US criteria with narrower screening criteria reduced the workload by 66.5%, only one infant with type I ROP that required treatment was missed in 2011. Therefore, the UK criteria may be more appropriate for screening ROP in our NICU.

Since the initiation of the ROP screening guideline by the Ministry of Health of China in 2004, ROP screenings are required to be conducted in infants with GA ≤ 34 weeks and/or BW ≤ 2000 g or who have had prolonged use of oxygen therapy. Since then, ROP studies have been conducted in different regions in China (Table 5). Most of these studies have been published in Chinese journals. These studies reported a great variation in the ROP incidence (range, 10.8%–41.6%). Although these studies varied by screening criteria, screening methods, and had a relatively small sample size, they have shown that the ROP incidence is associated with socioeconomic conditions and child healthcare. For example, in the developed regions such as Beijing, Shanghai, Guangdong, Zhejiang, and Shandong, the ROP incidence is relatively low (range, 10.8%–17.8%). Consistent with these reports, the incidence rate of the present study was 11.9% (356/2997 infants) based on the ROP screening guideline initiated by the Ministry of Health of China in 2004. However, in less developed regions such as Jiangxi, Henan, Hebei, Shanxi, and Qinghai, the ROP incidence is much higher (range, 20.9%–49.9%) despite a range of wider screening criteria being applied. In addition, the incidence rate of type I ROP needing treatment is also higher in the less developed regions than in the developed regions. In addition, larger and older premature infants are more common in the less developed regions. Because the incidence of ROP varies greatly between different regions in China, uniform ROP screening criteria may not be appropriately applied for all regions across China. In the present study, ROP was identified in 356 (11.9%) of 2997 premature infants with GW ≤ 34 weeks and BW ≤ 2000 g. Of the 356 infants with ROP, 59 (2.0%) of them underwent laser photocoagulation or intravitreal injection of VEGF antibodies. The incidence rate of ROP and those requiring treatment reported in this study was lower than that (17.6% and 6.8%, resp.) observed in Shanghai during the same period [12]. In addition, we found that the incidence of ROP declined between 14.5% in 2009, 11.1% in 2010, and 9.5% in 2011, and the incidence of severe ROP declined between 2.1% in 2009, 2.0% in 2010, and 1.7% in 2011. The decline in the yearly incidence of ROP is most probably associated with the reduction of supplemental oxygen therapy, since the mean time for continuous oxygen therapy declined from 5.2 days in 2009 to 3.4 days in 2011. That may mainly benefit from the increased use of noninvasive ventilation and surfactant supplement. However, in this study the incidence rate of ROP in infants with BW ≤ 1500 g was 25.2% (207/820), and the incidence rate obviating treatment in all ROP infants was 16.6% (59/256), which was higher than that (20.3%, and 9.5%, resp.) from 1996 to 2000 in the US [26].

In this study, we evaluated different ROP screening criteria based on examination of 2997 premature infants in our NICU. If we adopted the criteria with GA ≤ 34 weeks and BW ≤ 2000 g, 987 infants did not require screening. This would reduce the screening number by 32.9%. Only two infants with type I ROP that was required for treatment were missed in 2010. If we adopted the screening criteria with GA ≤ 33 weeks and/or BW ≤ 1750 g, 588 infants were not required to be screened, resulting in reduction of the screening number by 19.6%. Only one infant needing treatment was missed in 2009. According to the UK criteria (GA ≤ 32 weeks and/or BW ≤ 1500 g), 1222 infants were not required to be screened, thus reducing the screening number by 40.8%. Although 53 ROP infants were missed including three infants with type I ROP, none of infants with type I ROP needing treatment was missed in 2011. Although the US criteria (GA ≤ 30 weeks and/or BW ≤ 1500 g) could reduce the screening number by 66.5%, 118 ROP infants were missed including one infant with type I ROP needing treatment who was missed in 2011. Therefore, the UK criteria may be more appropriate for screening ROP in our NICU. However, a prospective NICU-based study in Shanghai showed that type I ROP needing treatment was missed if the UK and US criteria were adopted, suggesting that the UK and US criteria may not be suitable for ROP screening in China [12]. Narrower criteria with GA ≤ 33 weeks and/or BW ≤ 1750 g may be more appropriate for ROP screening in the NICU in Shanghai [12]. Therefore, the ROP screening criteria initiated by the Ministry of Health of China in 2004 are too wide for screening ROP in the NICU in developed regions in China such as Beijing and Shanghai. Even in developed regions, uniform criteria for screening ROP may not be applied to different NICU. Currently, the best ROP screening criteria should be adopted based on the specific conditions of each NICU but this may have logistical issues. Furthermore, intraregion comparison of ROP incidence rates would be much harder to do.

This study has some limitations. First, this is a single center study conducted in Beijing, the capital of China, with better health care system than other regions in China. Future multicenter epidemiologic studies with a large sample size in different areas are required for developing suitable ROP screening criteria in China. Second, this study only included infants for 3 years, and we found that the UK criteria were only appropriate for screening ROP in our NICU in 2011. Further studies with longer periods should be performed to confirm whether the UK criteria are appropriate for screening ROP in China.

## Figures and Tables

**Table 1:** The baseline characteristics of ROP and non-ROP infants.

	ROP (*n* = 356)	Non-ROP (*n* = 2641)	*p* value
Sex: male/female	197/159	1549/1092	0.252
Multiple births (%)	50 (14.0)	403 (15.3)	0.213
Supplemental oxygen	314 (88.2)	1608 (60.9)	0.000
GA, mean ± SD, wk	30.47 ± 1.99	32.09 ± 1.82	0.000
BW, mean ± SD, kg	1.48 ± 0.37	1,86 ± 0.43	0.000
Asphyxia at birth (%)	87 (24.4)	421 (15.9)	0.001
Septicemia (%)	44 (12.4)	103 (3.9)	0.000
BPD (%)	67 (18.8)	56 (2.1)	0.000
Blood transfusions (%)	100 (28.1)	365 (13.8)	0.000
NRDS (%)	203 (57.0)	760 (28.8)	0.000
Apnea	37 (10.4)	103 (3.9)	0.000
Assisted ventilation (%)	225 (63.2)	798 (30.2)	0.000

**Table 2:** The incidence rate of ROP with different stages according to gestational age and birth weight.

	Non-ROP *n* (%)	Stage 1 ROP *n* (%)	Stage 2 ROP *n* (%)	Stage 3 ROP *n* (%)	Treated ROP *n* (%)	Total infants *n*
GA, (W)						
≤26	8 (66.7)	0 (0)	2 (16.7)	2 (16.7)	3 (25)	12
27-28	100 (64.1)	20 (12.8)	26 (16.7)	10 (6.4)	18 (11.5)	156
29-30	396 (76.9)	41 (8.0)	65 (12.6)	13 (2.5)	20 (3.9)	515
31-32	904 (88.5)	43 (4.2)	62 (6.1)	12 (1.2)	15 (1.5)	1021
33-34	1139 (95.2)	34 (2.8)	22 (1.8)	1 (0.1)	3 (0.3)	1196
35-36	94 (96.9)	0 (0)	3 (3.1)	0 (0)	0 (0)	97

Total	2641 (88.1)	138 (4.6)	180 (6.0)	38 (1.3)	59 (2.0)	2997

BW, (g)						
≤1000	38 (54.3)	8 (11.4)	15 (21.4)	9 (12.9)	15 (21.4)	70
1001–1250	166 (66.9)	23 (9.3)	47 (19.0)	12 (4.8)	17 (6.9)	248
1251–1500	409 (81.5)	34 (6.8)	51 (10.2)	8 (1.6)	10 (2.0)	502
1501–1750	481 (86.8)	34 (6.1)	34 (6.1)	5 (0.9)	10 (1.8)	554
1751–2000	668 (93.3)	20 (2.8)	25 (3.5)	3 (0.4)	5 (0.7)	716
>2000	879 (96.9)	19 (2.1)	8 (0.9)	1 (0.1)	2 (0.2)	907

Total	2641 (88.1)	138 (4.6)	180 (6.0)	38 (1.3)	59 (2.0)	2997

**Table 3:** The incidence rate of ROP with different zones according to gestational age and birth weight.

	Non-ROP *n* (%)	Zone 1 ROP *n* (%)	Zone 2 ROP *n* (%)	Zone 3 ROP *n* (%)	AP-ROP *n* (%)	Total infants *n*
GA, (W)						
≤26	8 (66.7)	2 (16.7)	2 (16.7)	0 (0)	3 (25.0)	12
27-28	100 (64.1)	12 (7.7)	34 (21.8)	10 (6.4)	8 (5.1)	156
29-30	396 (76.9)	14 (2.7)	77 (15.0)	28 (5.4)	7 (1.4)	515
31-32	904 (88.5)	11 (1.1)	58 (5.7)	47 (4.6)	1 (0.1)	1021
33-34	1139 (95.2)	3 (0.3)	28 (2.3)	26 (2.2)	0 (0)	1196
35-36	94 (96.9)	0 (0)	2 (2.1)	1 (1.0)	0 (0)	97

Total	2641 (88.1)	42 (1.4)	201 (6.7)	112 (3.7)	19 (0.6)	2997

BW, (g)						
≤1000	38 (54.3)	12 (17.1)	18 (25.7)	2 (2.9)	8 (11.4)	70
1001–1250	166 (66.9)	12 (4.8)	55 (22.2)	15 (6.0)	5 (2.0)	248
1251–1500	409 (81.5)	7 (1.4)	58 (11.6)	28 (5.6)	2 (0.4)	502
1501–1750	481 (86.8)	7 (1.3)	34 (6.1)	31 (5.6)	2 (0.4)	554
1751–2000	668 (93.3)	2 (0.3)	23 (3.2)	23 (3.2)	2 (0.3)	716
>2000	879 (96.9)	2 (0.2)	13 (1.4)	13 (1.4)	0 (0)	907

Total	2641 (88.1)	42 (1.4)	201 (6.7)	112 (3.7)	19 (0.6)	2997

**Table 4:** Evaluation of different screening criteria for ROP.

Screening criteria	Infants meeting criteria	Infants not required for screening	Screening number reduction (%)	Missed ROP infants, *n*	Missed infants with type I ROP	Missed infants with ROP (type I ROP) in 2009	Missed infants with ROP (type I ROP) in 2010	Missed infants with ROP (type I ROP) in 2011
GA ≤ 34 W and BW ≤ 2000 g	2010	987	32.9	31	2	17 (0)	11 (2)	3 (0)
GA ≤ 33 W and/or BW ≤ 1750 g	2409	588	19.6	18	1	14 (1)	3 (0)	1 (0)
GA ≤ 32 W and/or BW ≤ 1500 g (UK)	1775	1222	40.8	53	3	33 (1)	17 (2)	3 (0)
GA ≤ 30 W and/or BW ≤ 1500 g (US)	1005	1992	66.5	118	10	63 (4)	42 (5)	13 (1)

**Table 5:** Recent ROP screening reports in China.

Author	Province	Screening period	Study type	Screening criteria	Number of ROP patients	Incidence of all ROP (%)	Treated-ROP rate (%)	GDP per head in 2013 ($)
Shenzhen ROP Cooperative group [14]	Shenzhen	2004.1~2013.6	Retrospective Multiple center	GA ≤ 34 W and/or BW ≤ 2000 g	9100	12.49 2004.1–2008.12 14.64% 2009.1–2013.6 11.47%	4.99 2004.1–2008.12 6.52% 2009.1–2013.6 4.26%	9474.66

Beijing ROP Epidemiology Study group [15]	Beijing	2005.1~2005.12	Retrospective Multiple centers	GA ≤ 34 W and/or BW ≤ 2000 g	639	10.8	3.6	15216.31

Xu et al. [12].	Shanghai	2010.1*～*2012.12	Prospective Multiple center	GA ≤ 34 W or BW ≤ 2000 g or any infant ventilated for at least 1 week or received supplemental oxygen for more than 30 days	2825	17.8	6.8	14652.98

Jin et al. [16].	Zhejiang	2005.3*～*2008.11	Retrospective Multiple centers	GA ≤ 34 W and/or BW ≤ 2000 g	1225	10.8	0.3	11075.57

Lin et al. [17]	Guangdong	2005.1*～*2008.12	Retrospective Single center	BW ≤ 2000 g	522	12.1	2.1	9474.66

Cheng et al. [18]	Shandong	2007–2010	Retrospective Single center	GA ≤ 37 W	773	11.13	4.1	9117.04

Qiu et al. [19]	Jiangxi	2005.1*～*2008.4	Retrospective Single center	GA ≤ 37 W and/or BW ≤ 2500 g	182	20.9	Unknown	5140.4

Chen et al. [20].	Hebei	2006.12*～*2007.8	Retrospective Single center	GA ≤ 36 W and/or BW ≤ 2000 g	398	30.15	2.26	6270.67

Ju et al. [21]	Qinghai	2005.7~2008.6	Retrospective Single center	GA ≤ 34 W and/or BW ≤ 2000 g	240	23.3	5.4	5918.86

Zhao et al. [22].	Shanxi	2005.1~2008.12	Retrospective Single center	GA < 37 W	572	49.6	11.5	6903.05

Zhang [23].	Henan	2006.12~2009.10	Retrospective Single center	GA < 37 W	420	26.4	2.5	5520.01
